# Supplementary material for: Individualized Dosage of Tacrolimus for Renal Transplantation Patients Based on Pharmacometabonomics
Source: Molecules. 2022 May 30;27(11):3517. doi: 10.3390/molecules27113517 (PMC9182099; doi:10.3390/molecules27113517)
Supplement: Supplementary file 1 [file molecules-27-03517-s001.zip › molecules-1710835-supplementary.pdf]

Supplementary Material

Individualized dosage of tacrolimus for renal transplantion patients based on pharmacometabonomics

Xiaoying He[a], Xi Yang[a], Xiaoting Yan[a], Mingzhu Huang[*a], Zheng Xiang[*b], Yan Lou[*a]

a Zhejiang Provincial Key Laboratory for Drug Clinical Research and Evaluation, Department of Clinical Pharmacy, The First Affiliated Hospital, College of Medicine, Zhejiang University, 79 QingChun Road, Hangzhou, Zhejiang 310000, People's Republic of China

[b] Zhejiang University City College, Hangzhou, Zhejiang 310000, People's Republic of China

* Correspondence: yanlou@zju.edu.cn; Tel.: +8657187236871

The raw non-targeted metabolomic MS data was as follows：

NEG.I.csv POS.M.csv POS.I.csv NEG.M.csv
